# Anti-Adhesive Effect of Porous Polylactide Film in Rats

**DOI:** 10.3390/polym13060849

**Published:** 2021-03-10

**Authors:** Kyu Jin Chung, Youn Jung Kim, Tae Gon Kim, Jun Ho Lee, Yong-Ha Kim

**Affiliations:** 1 Department of Plastic & Reconstructive Surgery, College of Medicine, Yeungnam University, Daegu 42415, Korea; kimtg0919@daum.net (T.G.K.); junojunho@gmail.com (J.H.L.); kimyon@ynu.ac.kr (Y.-H.K.); 2 Department of Applied Chemistry, Andong University, Andong 36729, Korea; sanso300@hanmail.net

**Keywords:** tissue adhesions, barrier, polylactide

## Abstract

Excessive adhesion between tissues on a significant area can cause the development of disorders, cosmetic problems, and ileus. Methods for preventing adhesion include the use of drugs and anti-adhesion barriers for physical blocking. In this study, the adhesion prevention effect of polylactide film in porous form was analyzed. A porous polylactide film was manufactured using a molecular weight of at least 100,000. To generate porosity, 98% methylene chloride and 95% ethyl alcohol were used as solvents. The thickness, surface, and internal pore shape of film were investigated. The crystal structures and melting temperature of film were measured. In the rat model, the presence and severity of adhesion were then analyzed. The thickness of the film ranged from 10 to 20 µm. The surface of the film contained pores with diameters of less than 10 µm. Partial crystallinity appeared from 15° to 20°, but the structure was amorphous overall. In the rat cecum abrasion model, adhesion occurred in 3 of the 13 rats in the polylactide experimental group, representing a 23.1% incidence rate. There were statistically significant differences in the severity of adhesion. The use of porous polylactide films can reduce the incidence of adhesion.

## 1. Introduction

Adhesion refers to the mechanism of binding between tissues that are separated from each other. Adhesions developing in the tendons of the arms and hands can lead to the development of disorders, such as restricted range of motion. Additionally, excessive adhesion between the skin and subcutaneous tissue can lead to cosmetic problems. Furthermore, adhesions occurring in the abdomen can cause ileus that is likely to develop into a life-threatening problem [1,2]. Therefore, adhesion should be either prevented or delayed.

Surgery-related adhesion can occur because of several causes, including tissue damage, ischemia, and foreign body reactions [3,4]. The dissolution of fibrin, which is a fibrous protein associated with thrombus formation, is suppressed and locked-in based on interruptions caused by the influence of surgery or trauma. Additionally, cellular structures with vascular distributions are formed after these events and can cause adhesion [5].

Methods for preventing adhesion include the use of drugs that inhibit the factors acting in the pathogenesis mechanisms of adhesion and the use of anti-adhesion barriers for physical blocking [6,7]. Drugs used for this purpose include anti-inflammatory agents, anti-coagulants, fibrinolytic agents, and antibiotics. The effectiveness of these drugs remains clinically unverified, and their action is not continuous when applied locally; thus, they cannot effectively prevent adhesion in all cases [8].

Anti-adhesion barriers can be divided into instillates and adhesion blockers. Instillates are available as gels manufactured from heparin, carboxymethyl cellulose, and hyaluronic acid and as solutions such as icodextrin and dextran-70 [9,10,11]. However, instillates are easily dislocated from damaged tissues and frequently fail to function effectively for adhesion prevention.

Adhesion blockers are available in the form of films manufactured from oxidized cellulose, hyaluronic acid, and carboxymethyl cellulose combination agents, as well as 70:30 poly(l-lactide-*co*-d,l-lactide), polyethylene glycol, and polylactide combination agents [12,13]. A 70:30 poly(l-lactide-*co*-d,l-lactide) film manufactured with polylactide acting as an adhesion prevention agent has been reported to prevent adhesion in various animal models and is widely used in clinical settings [14]. Such a film can effectively inhibit the adhesion of dura [15]. In this study, polylactide was manufactured as a porous film. The form of the manufactured film was identified, and the adhesion prevention effect of the film was analyzed.

## 2. Materials and Methods

### 2.1. Manufacturing Porous Film Using Polylactide

A porous polylactide film was manufactured according to the method described in Published Patent No. KR101082935B1 [16]. The polylactide (Human C&D, Daegu, Korea) used in this study had a molecular weight of at least 100,000. Additionally, 98% methylene chloride and 95% ethyl alcohol were used as solvents to generate porosity. Following the preparation of a 10% polylactide solution by completely dissolving 100 g of poly(l-lactide) in 900 g of methylene chloride, 15 mL of ethyl alcohol was added to 25 mL of the solution to prepare a mixed solution. A 100 mm × 100 mm polyethylene plate was coated with the mixed solution and dried for 4 h at room temperature (21 °C), followed by the removal of methylene chloride and ethyl alcohol. To ensure the complete removal of residual solvents, the plate was dried for an additional 10 min under a 90 °C hot air dryer. The porous polylactide film was then separated from the polyethylene plate. The separated porous polylactide film was analyzed to determine its morphology using a scanning electron microscope and then used in an animal study.

### 2.2. Analysis of the Physicochemical Properties of Film

#### 2.2.1. Investigation of the Thickness, Surface and Internal Pore Shape of Film

For observation using a scanning electron microscope (JSM-6300, Jeol, Tokyo, Japan), the front, back, and sides of the films were fixed on carbon tapes and coated with gold after cutting the films into 5 mm × 5 mm squares. The coating conditions are summarized as follows: a vacuum (10^−3^ mmHg) was applied to remove moisture and air, and plasma was then generated to coat the gold for 50 s after introducing argon gas. The coated film was observed at 100×, 500×, and 1000× magnification using a scanning electron microscope. The pore distributions formed on the front, back, and inside of the film, as well as the thickness of the side of the film, were investigated.

#### 2.2.2. Analysis of the Crystal Structures of Film

Crystal structures were analyzed using an X-ray diffractometer (Ultima IV, Rigaku, Tokyo, Japan) under conditions of 35 KV, 20 mA, scan range from 5° to 60°, scan step of 0.05°, and scan rate of 2°/min.

#### 2.2.3. Melting Temperature Measurement of Film through Thermal Analysis

The melting points of the samples were determined using a differential scanning calorimeter (DSC 200 Phox, Netzsch, Selb, Germany) over a temperature range of 20 to 300 °C at a heating rate of 5 °C/min in an air atmosphere. To determine the thermal and mass changes, samples were analyzed through thermogravimetric analyzer (TG-8120, Rigaku, Japan) over a temperature range of room temperature to 700 °C at a heating rate of 5 °C/min in an air atmosphere.

### 2.3. Analysis of Adhesion Prevention Effect in an Animal Study

**Figure 1:**
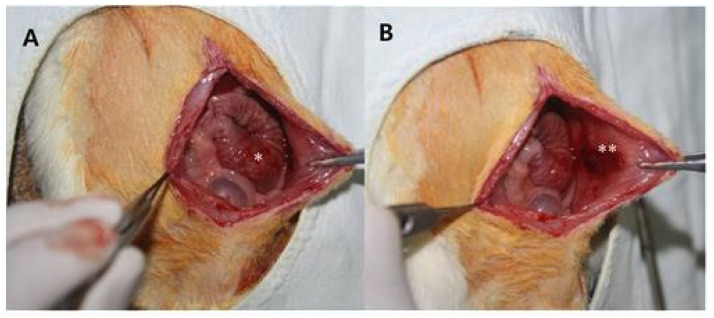
Photograph of the surgical technique. (**A**) The cecum was abraded with abrasive paper until an area of 100 mm^2^ was deserosalized, as evidenced by punctate bleeding. (**B**) The abdominal wall was incised, and a 100 mm^2^ defect was created by removing the peritoneum, including some muscle fibers. Note: * highlights the abraded cecum, and ** highlights the damaged abdominal wall.

**Figure 2:**
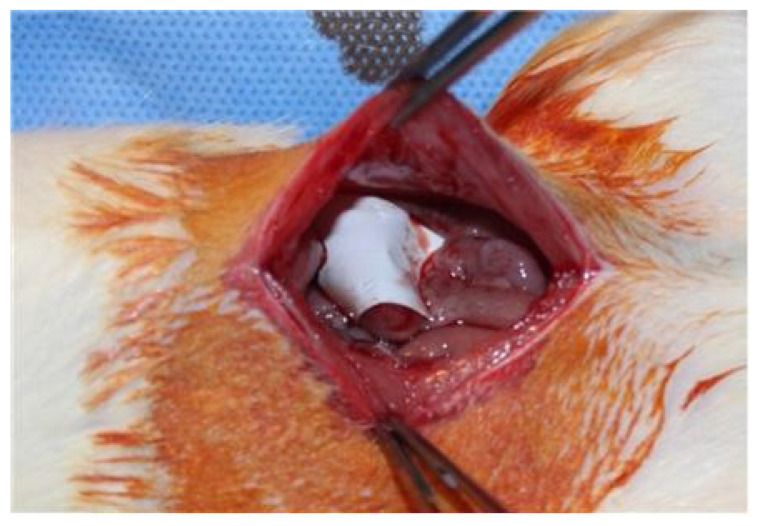
Application of a porous polylactide film (20 mm × 20 mm) to the injured cecum.

An animal study was performed in compliance with the Ethical Standards (Approval No. YUMC-AEC2014-004) of the Ethical Committee for Animal Study at our university. We first prepared 20 mm × 20 mm porous polylactide films and selected 12-week-old rats weighing 300 g for our animal study. For anesthesia, a mixture of 40 mg/kg of ketamine hydrochloride (Ketara^®^, Yuhan Corporation, Seoul, Korea) and 3 mg/kg of xylazine (Rumpun^®^, Bayer, Korea) was dosed to the rats via intramuscular injection. The rats were placed in supine position, the hair on the operation site was removed, and the site was disinfected using a povidone–iodine solution. A 40 mm incision was then made along the median line of the abdomen. After making a 40 mm incision along the exposed peritoneum, the intraperitoneal cecum was abraded using abrasive paper (No. 800, Daesung Abrasive Co., Seoul, Korea) until the formation of a petechia (minor bleed from capillary vessels) with a size of 10 mm × 10 mm. A defect of the same size was then created by removing the peritoneum and some muscular fibers from the wall of the site in contact with the cecum. Additionally, two parallel incisions and two vertical incisions were made on the peritoneum on the wall side. In 16 rats (control group), the peritoneum and skin were sutured using 5-0 vicryl and 5-0 nylon, respectively, with no insertions. In 16 rats (experimental group), porous polylactide films were attached to the surface of the peeled serous membrane of the cecum, and suturing was performed in the same manner as in the control group (Figure 1 and Figure 2).

### 2.4. Visual Observation of Adhesion Severity 

The rats were euthanized three weeks after the operations, and U-shaped laparotomies were performed. Visual observation was performed to determine the severity of adhesion between the cecum and peritoneum, followed by detachment. After assessing the presence/absence of adhesion, the severity of adhesion was classified according to the following numeric grades for assessment: 0 = absence of adhesion, 1 = adhesion in the form of a membrane that is easily separable through non-incisional detachment, 2 = minor to moderate adhesion separable by incision, 3 = moderate to severe adhesion that is difficult to separate or inseparable via incisional detachment (Table 1).

### 2.5. Statistical Analysis

Statistical analysis was performed using the SPSS 17.0 (IMB SPSS Statistics, Armonk, NY, USA) software. The presence/absence of adhesion between the two groups was analyzed using a chi-squared test, whereas the severity of adhesion was analyzed using a Mann–Whitney test. A *p*-value less than 0.05 was considered to be statistically significant.

## 3. Results

### 3.1. Physicochemical Properties of Film

#### 3.1.1. Thickness, Surface, and Internal Pore Shapes of Film

**Figure 3:**
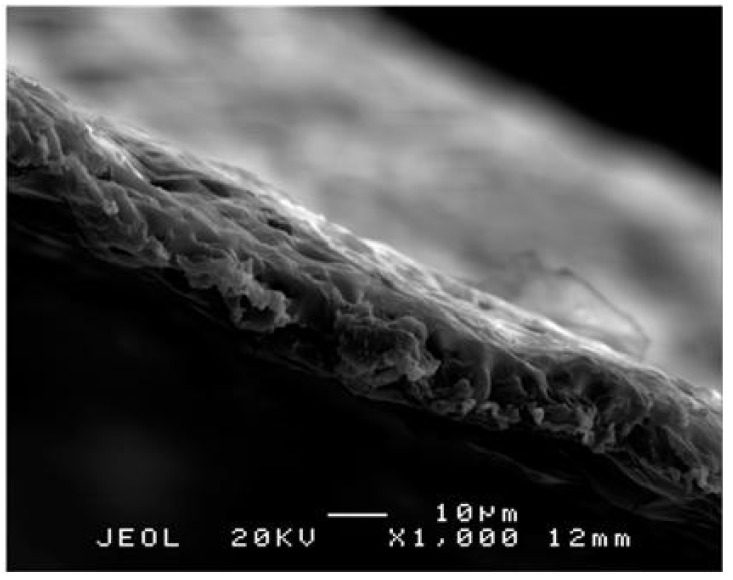
Scanning electron microscope image of a porous polylactide film. The thickness of film ranges from 10 to 20 µm. Note: Scale bar = 10 µm.

**Figure 4:**
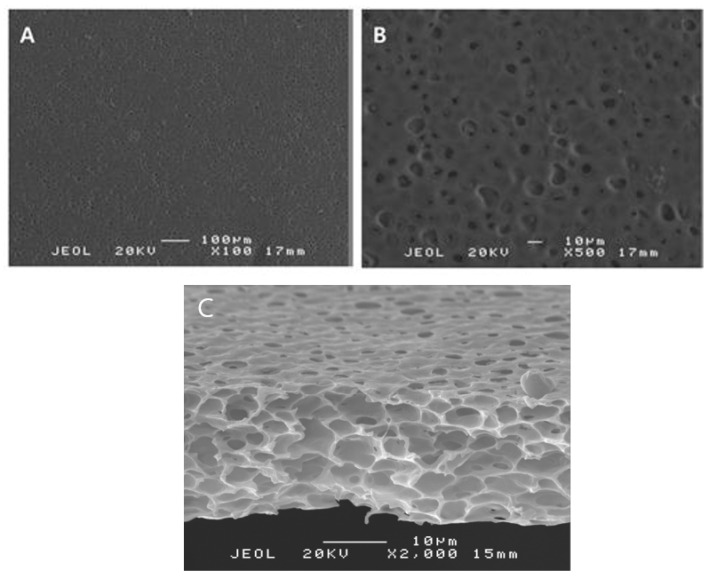
Scanning electron microscope images of the surface and inner morphology of a porous polylactide film. The surface of the film contains pores with diameters of less than 10 µm. (**A**) 100× magnification. (**B**) 500× magnification. (**C**) Interior of the film contains interconnected pores like a honeycomb.

The thicknesses of the porous polylactide film ranged from 10 to 20 µm, and pores with diameters of ≤10 µm were irregularly distributed on their surfaces. The interiors of the film contained honeycomb-shaped pores (Figure 3 and Figure 4).

#### 3.1.2. Crystal Structures of Film

**Figure 5:**
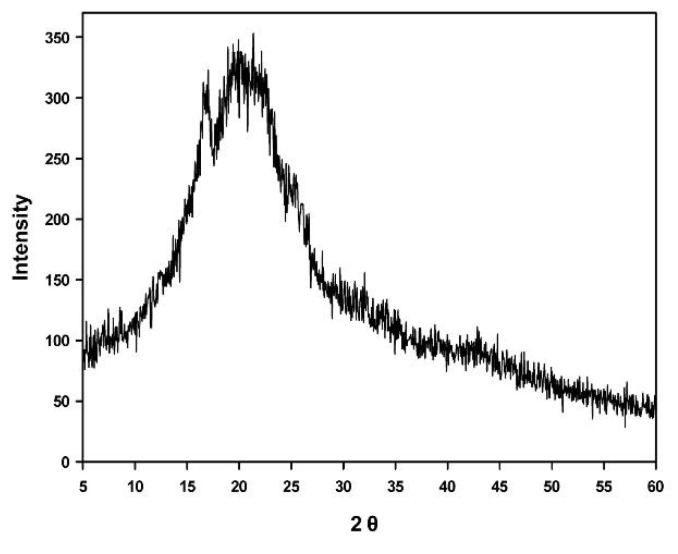
Crystallinity of a film measured using a powder X-ray diffractometer. Partial crystallinity appears from 15° to 20°, but the structure is amorphous overall.

The crystal structures of the film were analyzed using an X-ray diffractometer, revealing an amorphous structure overall, even though partial crystallinity appeared between 15° and 20°, suggesting that the film was amorphous when it was generated as a porous film using polylactide (Figure 5).

#### 3.1.3. Melting Temperature of Film

**Figure 6:**
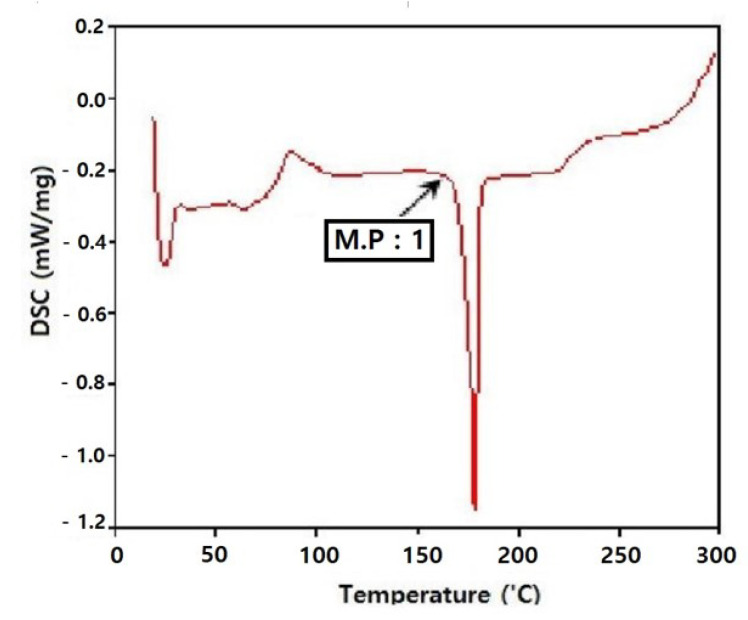
Melting analysis of films using a differential scanning calorimeter. The melting point (M.P:1) of the film is 175 °C.

**Figure 7:**
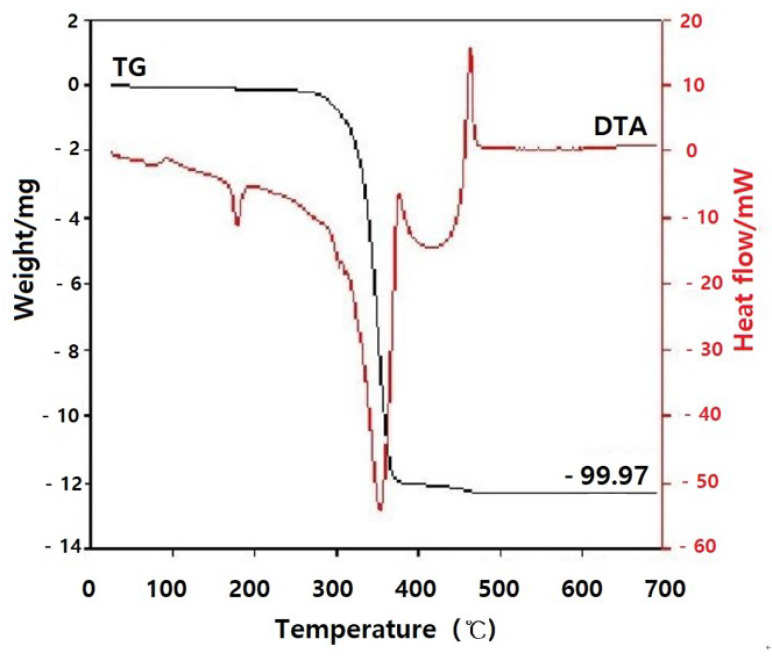
Thermal analysis system. Ash fraction of the film was less than 0.05% (TG, thermal gravity). The film was volatilized over the temperature range of 300 to 400 °C (DTA, differential thermal analysis).

The melting point of the porous polylactide film was analyzed to be 175 °C using differential scanning calorimeter analysis. According to the thermogravimetric analyzer, the conditions were as follows: 99.97% of the volatile substances, 0.05% of ash, and 300 to 400 °C of the volatile temperature (Figure 6 and Figure 7).

### 3.2. Animal Study

In total, 2 (12.5%) of the 16 rats in the control group and 3 (18.8%) of the 16 rats in the test group died during the study period. However, no statistical differences in mortality were observed between the two groups. All five of the aforementioned rats died within three days of the operations, and cecal perforations were observed at the time of necropsy. No residual films were observed in the test group during laparotomies performed three weeks after the operations.

**Figure 8:**
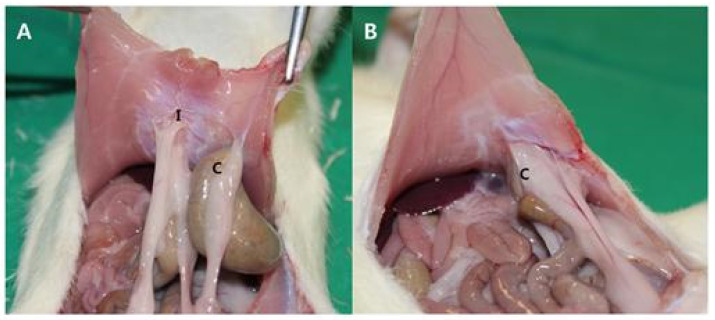
Photographs of the control group. (**A**) Adhesions were thick and extensive in 13 cases. (**B**) Moderate to severe adhesions were observed between the cecum and the peritoneum even while pulling peritoneum. Note: “C” represents cecum, and “I” represents incision site.

**Figure 9:**
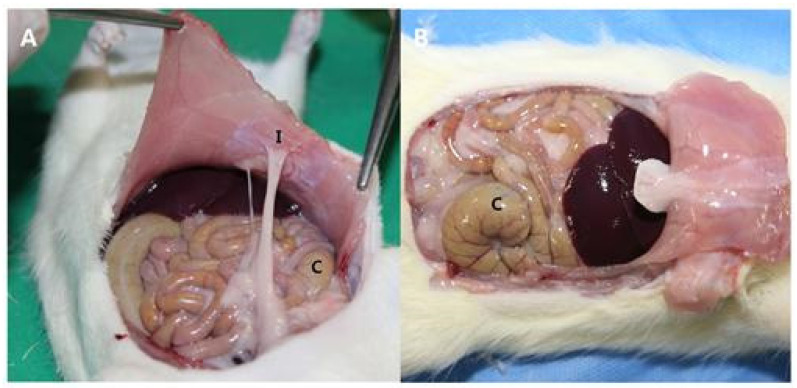
Photographs of the test group. (**A**) Adhesions between the incision in the abdominal wall and other tissues can be observed. (**B**) There are fewer adhesions between the cecum and abdominal wall. Note: “C” represents cecum, and “I” represents incision site.

**Figure 10:**
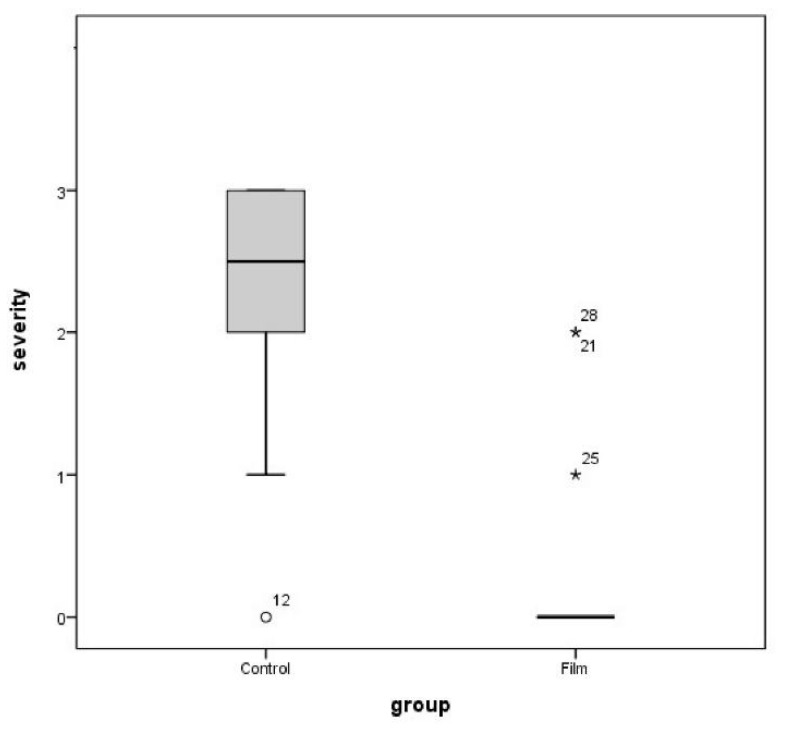
Graphical representations of the adhesion severity scores of the two groups. Median (line within the box), interquartile range (box), and range (error bars) are shown. The differences are statistically significant between the two groups (Mann–Whitney U-shaped test, *p* < 0.001). Note: 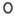 represents no adhesion cases in the control group (rat number 12). 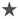 represents adhesion cases in the test group (rat numbers 21, 25, and 28).

Adhesion between the cecum and peritoneum occurred in 13 of the 14 rats in the control group, representing a 92.9% incidence rate (Figure 8). In contrast, adhesion only occurred in 3 of the 13 rats in the test group, representing a 23.1% incidence rate (Figure 9). These results represent statistically significant differences between the two groups (chi-squared test, *p* < 0.001) (Table 2). Regarding the severity of adhesion, in the control group, grades 0, 1, 2, and 3 were represented by 1, 1, 5, and 7 rats, whereas in the test group, these grades were represented by 10, 1, 2, and 0 rats, respectively. Statistically significant differences were calculated between the severities of adhesion in the test and control groups (Mann–Whitney test, *p* < 0.001) (Figure 10).

## 4. Discussion

Polylactide has two chemical forms of l-lactide and d,l-lactide. Poly(l-lactide) is relatively strong with a long degradation time, whereas poly(d,l-lactide) is relatively weak and is characterized by a short degradation period. Depending on the proportions of these two chemical forms, the strength and degradation time of polylactide may vary.

Polylactide in the form of poly(l-lactide) was used in this study. The molecular weight of the polylactide used in this study for the synthesis process was 100,000 because polylactide with a molecular weight below 100,000 can easily be damaged. The greater the molecular weight, the greater the tensile strength of the film; however, its in vivo absorption time may be longer [16]. Additionally, a mixing ratio of polylactide below 10% in methylene chloride used as a solvent may lead to difficulty in pore formation. However, if the ratio exceeds 20%, the production of a homogenous mixture becomes difficult because of the increased viscosity. If the mixing ratio in ethyl alcohol as a solvent is less than 10%, no pore formation occurs on the surface or interior of the film, whereas if the ratio exceeds 100%, the precipitation of polylactide occurs in the mixed solution [16].

In our animal study, adhesion was induced by abrading the cecum and by creating defects on the abdominal wall. Abrasion was performed until petechia (minor bleeding from capillary vessels) was observed to generate sufficient adhesion, but we were cautious not to create intestinal perforations. Defects in the abdominal wall were made at sites in contact with the cecum. There are other methods that can achieve better adhesion, including suturing and fixation of the cecum and abdominal wall or creating additional abrasions on the small intestine [17]. The goals of this study were to determine the effects of a polylactide film for adhesion prevention and to determine the flexibility and usability of such films on round organs. In addition, we aimed to identify any problems that might be caused by the shifting of film after wrapping the site of damage to the cecum with the porous polylactide film. Additionally, because the adhesion of other intraperitoneal organs and tissues to the abdominal wall was induced by creating a defect with dimensions of 10 mm × 10 mm on the abdominal wall and by making two incisions in parallel and vertical directions on the peritoneum, we aimed to determine the effects of film application to the cecum on adhesion between the cecum and abdominal wall.

Polylactide films prevent adhesion by forming a physical barrier between adjacent tissues during the period in which adhesion is likely to occur. Peritoneal tissues exhibit fibrin deposition within 12 h after being damaged, and the formation of new mesothelium begins after two to three days and is completed within seven to nine days [18]. According to Harris et al. [19], the first 36 h are critical for adhesion formation. In contrast, Gomel et al. [20] suggested that adhesion occurs between seven and ten days after an operation and that this period is an important period for the prevention of adhesion.

In this study, no remaining films were observed in the rats during laparotomies performed three weeks after the operations. Thirteen rats (all but one) in the control group exhibited adhesion of the cecum to the abdominal wall. In contrast, relatively minor adhesion of intraperitoneal tissues other than the cecum to the abdominal wall was observed in the test group. Based on these results, it can be concluded that our study model is suitable for induced adhesion. Regarding the severity of adhesion, the adhesion grades were significantly lower in the test group compared to the control group, suggesting that the porous polylactide films were sufficient as a physical barrier against adhesion. Only 3 of the 13 rats with film insertions exhibited adhesion between the cecum and abdominal wall. We hypothesize that adhesion may have occurred as the porous polylactide films were degraded by hydrolysis before the abdominal wall or cecum were healed from the induced damage, resulting in breakage of the films into pieces or shifting of the films.

The existing adhesion blockers such as 70:30 poly(l-lactide-*co*-d,l-lactide) films have been researched in various study models and are being used in clinical settings. Avital et al. [21] reported that a control group exhibited a 100% incidence rate of adhesion in rat models with cecum–abdominal wall adhesion, whereas a group with film insertions exhibited a 57.9% adhesion rate, representing a statistically significant difference. Regarding the degree of adhesion, 71.4% of the adhesion incidences in the control group and 10.5% in the test group were severe. Ersoy et al. [14] reported that in a rat model of cecum–abdominal wall adhesion, 100% adhesion occurred in the control group, whereas only 5 of 13 rats exhibited adhesion in the test group. Additionally, histological examinations revealed no statistically significant differences in fibrosis between the two groups, but there were significant differences in terms of inflammatory reactions. 

Comparative studies on polylactide films and other adhesion prevention membranes have yielded diverse results. In a study reported by Park et al. [4], a hyaluronan-based solution exhibited significantly lower adhesion grades compared to a control group, whereas polylactide films did not exhibit statistically significant results. The reported reason for the poor effectiveness of the polylactide films in terms of adhesion prevention was that suturing was required for the fixation of film, and if the number of sutures was insufficient, it was difficult to fix the film to the desired site. In contrast, if the number of sutures was too high, then suturing-induced adhesion would occur. In another study, the effects of three different adhesion prevention membranes were compared after performing mesh grafts for the correction of hernias in rats. Atelocollagen type 1, which is a hyaluronic acid–carboxymethyl cellulose complex adhesion prevention membrane, was effective at reducing adhesion to the mesh graft site, whereas polylactide films did not reduce adhesion compared to the control group. These results were explained based on the difficult manipulation of polylactide films [22]. When used in conjunction with mesh grafts, the effects of polylactide films as adhesion blockers were diminished [23]. In our study, the features of the porous polylactide films, including thinness and flexibility, provided easy manipulation and attachment to body-fluid-containing tissues. Therefore, sutures were unnecessary to prevent the shifting of the film, and suture-induced adhesion was reduced. 

## 5. Conclusions

The use of porous polylactide films can reduce the incidence of adhesion. In the future, it is expected that analgesic and antibacterial drugs, as well as extended-release mechanisms, could be utilized by adding various agents to the fine pores formed in polylactide films.

## Figures and Tables

**Table 1:** Adhesion scoring system.

Score	Adhesion Grade
0	No adhesion
1	Filmy adhesions that are easily separable through blunt dissection
2	Mild to moderate adhesions with free dissection
3	Moderate to severe adhesions with difficult dissection or no dissection

**Table 2:** Presence of adhesion in the rat cecum abrasion model.

	Number of Rats	Presence of Adhesion (Incidence Rate)
Control	14	13 (92.9%)
Porous polylactide film	13	3 (23.1%)

(chi-squared test, *p* < 0.001).

## Data Availability

Data available on request due to restrictions, e.g., privacy or ethical. The data presented in this study are available on request from the corresponding author.
